# Capillary Stalling: A Mechanism of Decreased Cerebral Blood Flow in AD/ADRD

**DOI:** 10.33696/neurol.2.048

**Published:** 2021

**Authors:** Reece Crumpler, Richard J. Roman, Fan Fan

**Affiliations:** Department of Pharmacology and Toxicology, University of Mississippi Medical Center, Jackson, MS 39216, USA

## Abstract

Alzheimer’s Disease (AD) and Alzheimer’s Disease-Related Dementias (ADRD) are debilitating conditions that are highly associated with aging populations, especially those with comorbidities such as diabetes and hypertension. In addition to the classical pathological findings of AD, such as beta-amyloid (Aβ) accumulation and tau hyperphosphorylation, vascular dysfunction is also associated with the progression of the disease. Vascular dysfunction in AD is associated with decreased cerebral blood flow (CBF). Impaired CBF is an early and persistent symptom of AD/ADRD and is thought to be associated with deficient autoregulation and neurovascular coupling. Another recently elucidated mechanism that contributes to cerebral hypoperfusion is capillary stalling, or the temporary arrest of capillary blood flow usually precipitated by a stalled leukocyte or constriction of actin-containing capillary pericytes. Stalled capillaries are associated with decreased CBF and impaired cognitive performance. AD/ADRD are associated with chronic, low-level inflammation, which contributes to capillary stalling by increased cell adhesion molecules, circulating leukocytes, and reactive oxygen species production. Recent research has shed light on potential targets to decrease capillary stalling in AD mice. Separate inhibition of Ly6G and VEGF-A has been shown to decrease capillary stalling and increase CBF in AD mice. These results suggest that targeting stalled capillaries could influence the outcome of AD and potentially be a target for future therapies.

## Introduction

Alzheimer’s Disease (AD) and Alzheimer’s Disease-Related Dementias (ADRD) are debilitating conditions highly associated with aging populations, especially those with comorbidities, such as diabetes and hypertension. AD is characterized by beta-amyloid (Aβ) accumulation, hyperphosphorylation of the tau protein (p-Tau), and cholinergic deficiency; however, potential treatment options focusing on these pathways have shown little efficacy [1,2]. It is known that cerebral hypoperfusion precedes the accumulation of Aβ and p-Tau deposits and is a harbinger for the later development of AD and ADRD and could be involved in disease progression [3,4]. Decreased cerebral blood flow (CBF) and focal hypoperfusion can be devastating considering the fact that the brain has a small energy reserve. Potential therapies improving cerebral perfusion have shown promise in improving cognitive function in AD. The mechanisms of cerebral hypoperfusion are poorly understood, but it is thought that both impaired autoregulation and neurovascular coupling may play a role [4–8].

## Poor Autoregulation Contributes to Decreased CBF

An inability to properly regulate vascular diameter in response to different stimuli can lead to deficits in CBF [9]. One potential mechanism for impaired autoregulation that is seen in AD patients is a change in vascular structure. AD is associated with excess collagen deposition and calcification in vessel walls which leads to increased wall thickness and decreased elasticity [3]. Structural changes in the vessels are associated with increased inflammation and reactive oxygen species (ROS) production. ROS production causes endothelial dysfunction, which impairs the vessel’s ability to dilate in response to decreases in arterial pressure, for example, during sleep. Vessel walls in AD patients can become overloaded with Aβ deposits in a phenomenon known as cerebral amyloid angiopathy (CAA), leading to decreased vessel elasticity and reduced autoregulation [10]. CAA can cause microhemorrhages and microinfarcts and is associated with an even greater decrease in CBF in AD patients [11].

## Neurovascular Uncoupling Contributes to Decreased CBF

Impaired neurovascular coupling is also associated with cerebral hypoperfusion. Neurovascular coupling, or functional hyperemia, is the process of an increase in neural activity leading to an increase in local CBF [12]. Functional hyperemia is necessary for normal brain function and is deficient in the elderly and AD patients [13]. This process is mediated by smooth muscle cells and pericytes in arterioles and capillaries, respectively [14–16]. Recent studies indicated that the hippocampus is more susceptible to neurovascular unit damage than the cortex [17,18]. Similar to the mechanisms behind impaired autoregulation, endothelial dysfunction due to increased ROS production contributes to neurovascular uncoupling [7,19]. Impaired neurovascular coupling was shown to be reversible in mice by the inhibition of mitochondrial ROS production [19]. The Nelson group found that a capillary endothelial cell inwardly rectifying K^+^ channel (Kir2.1) could potentially be implicated in the loss of neurovascular coupling [20–22]. They found that AD mice with deficient Kir2.1 channels had impaired functional hyperemia, which was rescued by a PIP2 analog [20].

## Capillary Stalling Contributes to Decreased CBF

Another elucidated mechanism that has been proposed to contribute to decreased CBF is capillary stalling, or the cessation of capillary blood flow predominantly caused by adhesion of circulating leukocytes [3]. Several mouse model studies have found an increase in the number of stalled capillaries, mostly due to leukocytes, in AD mice compared to wild type [3,23]. Brain capillary stalling also was found in anti-CD19 CAR T cell neuronal injury, suggesting long-term consequences of immunotherapy for cancer treatments in cerebral vascular function should be considered [24,25]. These stalled capillaries reduce CBF in the affected areas and contribute to the neurological deficits seen in AD. Multiple mechanisms have been theorized to contribute to capillary stalling, including increased inflammation, ROS, and endothelial cell dysfunction. One study examined the prevalence of capillary stalling in subcortical vascular dementia (SVaD) and its potential etiology [26]. They found that the number of stalled capillaries increased significantly in the SVaD group compared to the control and that certain areas were more prone to stalling than others [26]. The endothelial glycocalyx (EG) was also found to be less prominent in the stalled segments than in the normal segments, providing a possible mechanism contributing to capillary stalling. One possible explanation of how a thinned EG increased neutrophil adhesion and capillary stalling is that the EG normally promotes electrostatic repulsion of neutrophils since it is negatively charged. A thinned EG would therefore allow enhanced neutrophil access to the endothelium, promoting adhesion and stalling [26].

An additional mechanism that could potentially contribute to capillary stalling is pericyte dysfunction. Pericytes are contractile cells associated with capillaries that play a significant role in regulating CBF [14–16,27–29]. Pericyte loss and degeneration has been observed in neurodegenerative diseases such as AD and hypertension- and diabetes-related dementia [9,15,16,30–32]. Degeneration of pericytes can contribute to deficits in CBF. It was recently discovered that pericytes in both AD mice and AD patients were constricted, leading to decreased CBF [33]. The Zlokovic group found that inducing pericyte constriction in mice caused a decrease in RBC velocity in the corresponding capillaries [34]. They also found that some capillaries were stalled when the pericytes were constricted. Pericyte dysfunction in AD, therefore, causes capillary constriction and contributes to capillary stalling, leading to decreased CBF.

It is widely accepted that AD and ADRD are associated with increased levels of inflammation in the brain [35,36]. It is thought that one of the mechanisms behind this increased inflammation is a response to an increase in Aβ deposition [35]. Microglia show increased chemotaxis in response to Aβ, causing an influx of inflammatory cells in these areas within the brain [37]. Microglial activation increases the production of ROS and inflammatory cytokines such as IL-1β, IL-6, and TNF-α, all of which contribute to the progression of AD [38–40]. These inflammatory mediators that have been seen in AD also contribute to capillary stalling. An increase in inflammatory cytokines also increases the number of circulating leukocytes, which further exacerbates the number of stalled capillaries and therefore contributes to a decrease in CBF. Pro-inflammatory cytokines also promote upregulation of endothelial cell adhesion molecules, which promotes the adhesion of leukocytes to capillary walls. The Bracko group has discovered that leukocytes are the primary mediators of capillary stalling, with increased adhesion of neutrophils being the main contributor. Another group found that ischemic brain damage caused an increase in leukocyte capillary stalling, thought to be attributable to increased inflammation and upregulation of cell adhesion molecules [41].

## Potential Therapies Targeting Capillary Stalling

AD mice tend to have higher levels of endothelial cell adhesion molecule expression, leading to increased leukocyte rolling and transmigration into the brain parenchyma [36]. Recently, there have been a few discoveries of methods to reduce capillary stalling in AD mice and thus enhance CBF. One study found that the administration of an α_4_ integrin antibody caused decreased leukocyte adhesion and subsequently improved memory in AD mice [36]. This effect was presumed to be due to the decreased infiltration of leukocytes into the brain parenchyma, but it is possible that increased capillary blood flow due to decreased leukocyte adhesion also played a role. Another group found that the administration of an antibody against Ly6G, a neutrophil surface marker, led to an almost immediate decrease in the number of stalled capillaries and decreased the number of circulating neutrophils within several hours [23]. This resulted in an increase in CBF and an improvement in both working and short-term spatial memory in the AD rats treated with the antibody compared to those that were untreated. These results suggest that capillary stalling is not only reversible but is also a potential target for AD therapy.

ROS production is elevated in AD/ADRD patients and animal models [38,42]. As aging occurs, the balance between oxidative species and antioxidants is disturbed, leading to increased levels of ROS [42]. ROS are also implicated in diabetes, hypertension, and obesity, all of which are associated with the development of AD and ADRD. ROS production contributes to decreased vascular regulation in AD and also increases the expression of ICAM1 and VCAM1 on vascular endothelial cells [23]. These adhesion molecules interact with integrins on the surface of leukocytes, which can increase leukocyte adhesion and capillary stalling. Increased ROS production damages endothelial cells and results in a breakdown of the blood-brain barrier (BBB) [43]. This increase in vascular damage results in the production of angiogenic cytokines such as vascular endothelial growth factor (VEGF-A). VEGF-A has multiple effects throughout the vasculature and is increased in the blood of AD patients [43]. VEGF-A signaling has been shown to exert beneficial effects in AD mouse models, but it has also been implicated in capillary obstruction in mice with diabetic retinopathy [43]. In order to investigate this phenomenon further, Ali et al. administered anti-VEGF-A antibodies to AD mice, which resulted in a decrease in the stalled capillaries, an increase in capillary RBC flux, and a decrease in BBB permeability [43]. They also found that stalled capillaries tended to have decreased expression of the tight junction protein occludin compared to those that were flowing [43]. VEGF-A is known to decrease occludin expression via endothelial nitric oxide synthesis, which increases the permeability of vessels to allow for repair. These findings suggest that VEGF-A signaling plays a significant role in capillary stalling. However, because the pathways that involve VEGF-A are so numerous and diverse, inhibiting VEGF-A to prevent capillary stalling may provide more harm than benefit. VEGF-A administration has been shown to increase short-term memory and cognitive function in AD mice [44,45]. Ali et al. hypothesized that these contradictory effects of VEGF-A could be due to the fact that the anti-VEGF-A antibody crosses the BBB at an incredibly low rate, leading to an inhibition of VEGF-A signaling in the vasculature but having no effect on that of the brain parenchyma. If this were determined to be true, then inhibiting VEGF-A signaling via an IgG antibody could prove to be beneficial in AD patients as this inhibition would decrease capillary stalling but would have no effect on the positive aspects of VEGF-A signaling. In order to further investigate this theory, cognitive performance in anti-VEGF-A antibody-treated AD mice needs to be evaluated. The signaling pathways that involve VEGF-A are complex and necessitate more research to determine how they are involved in the progression of AD.

## Conclusion

**Figure 1:**
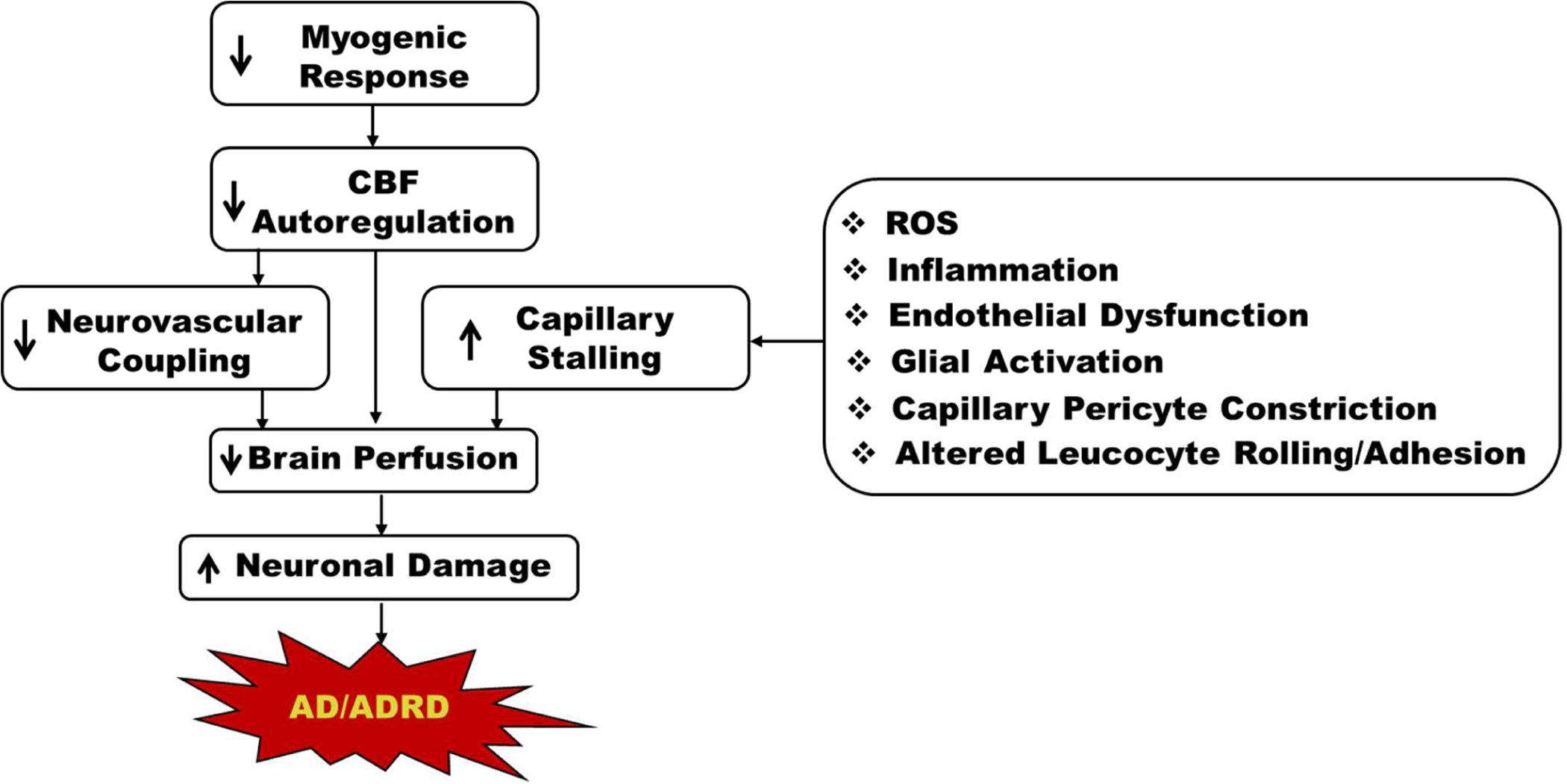
Summary of the essential mechanisms contributing to reduced CBF in AD/ADRD and various cellular mechanisms in capillary stalling.

Impaired autoregulation, deficient neurovascular coupling, and capillary stalling are just some of the mechanisms that contribute to cerebral hypoperfusion in AD/ADRD [3,43,46–48]. Capillary stalling is primarily due to increased adhesion of leukocytes to capillary endothelial cells but is also associated with increased pericyte constriction. This increased cell adhesion is precipitated by many mechanisms, including the chronic inflammation and increased ROS production that are seen in AD (Figure 1). Increased capillary stalling causes decreased CBF in the affected areas, which is an early and persistent symptom of AD. Recently, potential therapeutic targets to decrease capillary stalling in AD mice have been explored. The reversal of capillary stalling by these targets showed an increase in CBF in AD mice, suggesting that reversing capillary stalling could be beneficial in AD patients. While these therapeutic targets have been shown to reduce the number of stalled capillaries in AD mice, more research is needed to determine their potential efficacy in humans. Further study is also needed to determine how the reversal of capillary stalling impacts the progression of AD long-term.
